# Evaluation of the wear comfort of corporate identity clothes - the influence of water and soil repellency

**DOI:** 10.1186/2046-7648-4-S1-A93

**Published:** 2015-09-14

**Authors:** Edith Classen

**Affiliations:** 1 Hohenstein Institut für Textilinnovation gGmbH, Boennigheim, Germany

## Introduction

Corporate identity clothes (CI-clothes) are today more and more important for companies and their identity. One important aspect is the appearance of the clothes over the long lifetime. After several washing and wearing cycles the appearance of such clothes should only show low differences between new and worn clothes. To minimize the signs of use many CI-clothes have a water and soil repellent finishing avoiding contamination. This finishing shows often a negative influence on the comfort of the clothing. The aim of the German research project 16365N was to optimize water and soil repellent finishing in combination with high industrial wash ability and high comfort.

## Methods

In the project, typical fabrics of CI- clothes were finished with different auxiliaries for water and soil repellency as one and two side application. The different auxiliaries characterise the plurability of commercial available products (e.g. fluorocarbon dispersions (C6 and C8), fluorocarbon free dispersions, sol-gel based dispersions). The thermophysiological and skin sensorial comfort of the finished fabrics was determined by measurements with established devices: the Hohenstein skin model, a sweating guarded-hot plate and the five Hohenstein methods for the measurement of the skin comfort. For evaluation the thermophysiological comfort vote and the skin sensorial comfort vote was determined. At the end the total wear comfort was calculated.

## Results

The thermophysiological parameters of the finished fabrics of the fabric in new state and after finishing with different auxiliaries with variation of the finishing parameters and also the skin sensorial parameters were collected. The fabrics in origin state show in every case better thermophysiological properties than the finished fabrics. For all fabrics the total wear comfort of the finished products is lower than of the fabric in new state. The amount of the finishing detergents influenced the properties. In most cases high amounts of the finishing detergents degrade thermophysiological and skin sensorial properties. But with low amounts of the finishing detergents the water and soil repellency is not given.

## Discussion

The application of the water and soil repellent finishing on two sides leads to better thermophysiological comfort vote but also to a lower skin sensorial comfort vote. The one side application has the advantage of a better skin sensorial comfort but the thermophysiological comfort is lower caused by additional used thickener of the application. The influence of different water and soil repellent finishing to the total wear comfort are very similar independent on the chemistry of the finishing.

## Conclusion

The results of the research project show that the physiological comfort of CI-clothes can be influenced by the used water and soil repellent finishing. The application methods (one side coating or two side application via the foulard-process) have an important influence to the thermophysiological and skin sensorial comfort. The comfort vote system which is developed for all day clothes can be also used for CI-clothes but the additional functionalization lead to lower votes. At the moment all finishing auxiliaries for water and soil repellency degrade the thermophysiological and skin sensorial comfort. New developments of auxiliaries are necessary to improve also the wear comfort. In cases of CI-clothes the question to the needs of water and soil repellent finishing must be discussed to improve very easily the wear comfort of such clothes. In the case of PPE water and soil repellent finishing is often necessary and essential for the protection of the worker.
